# Diagnostic performance of Demirjian’s third molar maturation stages G, G1, and H for identifying the 18-year age threshold: a sex-specific study

**DOI:** 10.1007/s00414-026-03904-z

**Published:** 2026-07-06

**Authors:** Sudheer Babu Balla, Naga Madhuri Nagarakanti, Subramanyeswara Swamy Chinni, Waheeda Shahnaz, Manasa Bojji, Venkat Baghirath Pacha

**Affiliations:** 1 Department of Rural Clinical Sciences, La Trobe University, Bendigo, 3550 Australia; 2 Government Medical College, Mahbubnagar, Telangana India; 3 Clinical Practitioner, Chicago, USA; 4 SVS Institute of Dental Sciences, Mahabubnagar, Telangana India; 5 Malla Reddy Dental College for Women, Hyderabad., Telangana India; 6 Panineeya Institute of Dental Sciences & Research Center, Hyderabad, Telangana India

**Keywords:** Dental age estimation, Third molar, Demirjian method, Refined root stages, Age of majority, Diagnostic accuracy

## Abstract

Determining attainment of 18 years is a critical task in forensic age estimation. This study evaluated and compared the sex-specific diagnostic performance of conventional and refined third-molar maturation cut-offs, Demirjian stages G and H, and the refined stage G1 for identifying individuals aged ≥ 18 years in a South Indian population. Panoramic radiographs of 599 individuals aged 14–22 years were retrospectively assessed. Mandibular left third molars were staged using the conventional Demirjian system and the Solari and Abramovitch modification. Diagnostic performance was evaluated separately for males and females using sensitivity, specificity, predictive values, likelihood ratios, accuracy, and post-test probabilities. Intra- and inter-examiner agreement was assessed using kappa statistics. Third-molar maturation effectively discriminated attainment of 18 years in both sexes, with stage-dependent trade-offs between sensitivity and specificity. Stages G and above and stages G1 and above demonstrated high sensitivity and strong exclusion of adulthood when negative, whereas stage H showed higher specificity and greater confirmatory value when present, particularly among females. Diagnostic performance of the refined G1 stage was comparable to that of stage G, with modest improvement in specificity and post-test probability in females. Inter-examiner agreement was excellent for stages G and H but only moderate for G1, indicating reduced reproducibility for the refined stage. In conclusion, conventional Demirjian stages G and H remain reliable and reproducible indicators for assessing attainment of 18 years. Although the refined G1 stage offers increased developmental resolution and supports probabilistic interpretation, its forensic utility is constrained by reduced inter-observer agreement.

## Introduction

In forensic dental age estimation of living individuals, assessment during late adolescence and early adulthood remains particularly challenging. At this stage, most dental development has already been completed, while age-related regressive changes have not yet begun, resulting in a narrow biological window with limited age-informative markers [1, 2]. Consequently, only a limited number of age-informative biological markers are available, creating a narrow developmental window in which age estimation relies predominantly on third-molar maturation. This reliance on a single developmental indicator contributes to greater uncertainty than in earlier childhood or later adulthood. Within this age range, the third molars are of particular forensic relevance, as they are typically the only teeth still undergoing development, with formation extending into the mid-twenties [3]. For this reason, third-molar development is widely used in dental age estimation, especially to assess whether an individual has attained the legally significant age threshold of 18 years. The accuracy of such estimations depends not only on the method used to translate dental development into age but also on the precision with which developmental stages are identified and classified [4].

Against this background, third-molar-based dental age estimation has been extensively investigated. Mincer et al. [5] evaluated third-molar development in American Caucasians using the staging system proposed by Demirjian et al. [6], establishing a foundation for subsequent forensic applications. Since then, numerous studies have applied Demirjian’s third-molar developmental stages, particularly the late root stages, to support binary classification of individuals as younger or older than 18 years [7–11]. In addition, probability-based approaches using mandibular third-molar root development have been proposed to quantify the likelihood that an individual has attained 18 years of age, highlighting the practical utility of stage-based inference in forensic casework [12]. However, a recognised limitation of the original Demirjian A–H system is that developmental transitions occurring near apexification are subsumed within relatively broad stages, potentially limiting discriminatory power precisely at age ranges where legal decisions are most sensitive [13].

Building on these applications, it has been suggested that staging systems with fewer developmental categories are regarded as easier to apply because each stage encompasses a broader interval of tooth development. However, such broad stages span wide chronological age ranges and may therefore provide limited discriminatory power at legally relevant thresholds [14]. In contrast, staging systems that incorporate additional intermediate categories aim to capture finer developmental transitions over shorter time spans, potentially improving alignment with chronological age during late adolescence [15]. The refined root stages proposed by Solari and Abramovitch were introduced on this basis to enhance age discrimination near apexification [16]. As this modification is a direct extension of the widely used Demirjian classification, it offers an opportunity to evaluate whether additional developmental stages improve diagnostic performance. Although recent studies have begun to explore the diagnostic and reproducibility implications of the Solari and Abramovitch modification, evidence regarding the contribution of these intermediate stages to age discrimination remains limited [17, 18]. Therefore, the present study aimed to evaluate and compare the sex-specific diagnostic performance of conventional and refined third-molar maturation cut-offs, specifically stages G, G1, and H, for identifying individuals aged 18 years or older. By examining sensitivity, specificity, likelihood ratios, and post-test probabilities, this study sought to determine whether the inclusion of refined root stages improves the evidential reliability of dental age estimation in late adolescence.

## Materials and methods

### Study sample

Panoramic radiographs (orthopantomograms; OPGs) of 599 individuals with known chronological age and sex were retrospectively obtained from orthodontic clinics. The sample comprised adolescents and young adults of South Indian origin, aged between 14.0 and 21.99 years (Table 1). Chronological age was calculated to two decimal places as the difference between the date of birth and the date of radiographic exposure. For analytical purposes, individuals were grouped into one-year age categories (e.g., 14.0–14.99 years, 15.0–15.99 years, and so forth). Radiographs were excluded if they demonstrated severe dental caries affecting the third molars, previous endodontic treatment, pathological lesions involving the jaws, congenitally absent third molars, or insufficient image quality for reliable assessment. In addition, individuals with a reported history of endocrine disorders were excluded due to the potential influence on dental development.

**Table 1:** Age and sex distribution of the total sample n(%)

Age group (Years)	Males (%)	Females (%)
14- 14.99	40 (14.3)	42 (13.2)
15- 15.99	40 (14.3)	44 (13.8)
16- 16.99	40 (14.3)	35 (11)
17- 17.99	40 (14.3)	38 (11.9)
18- 18.99	37 (13.2)	40 (12.5)
19- 19.99	36 (12.9)	40 (12.5)
20- 20.99	28 (10)	50 (15.7)
21- 21.99	19 (6.8)	30 (9.4)
Total	**280 (100)**	**319 (100)**

The present study utilised OPGs derived from an existing anonymised dataset previously analysed and reported in earlier research [19, 20]. No additional radiographic acquisition or data collection was undertaken. All images were fully anonymised before analysis. For each case, chronological age, sex, and third-molar developmental stage were recorded against a unique identification number (UIN). Examiners were blinded to all personal and demographic information throughout the assessment process to minimise examiner bias. This study utilised OPGs from an existing anonymised database that had been previously analysed and reported in earlier research for which ethical clearance was obtained from the institutional ethics committee (PMVIDS&RC/IEC/OMFP/PR/0261 − 18). No additional data collection was undertaken for the present investigation. This study was conducted in accordance with the ethical standards established by the Declaration of Helsinki (Finland) and its subsequent amendments [21].

### Radiographic assessment and staging procedure

**Fig. 1:**
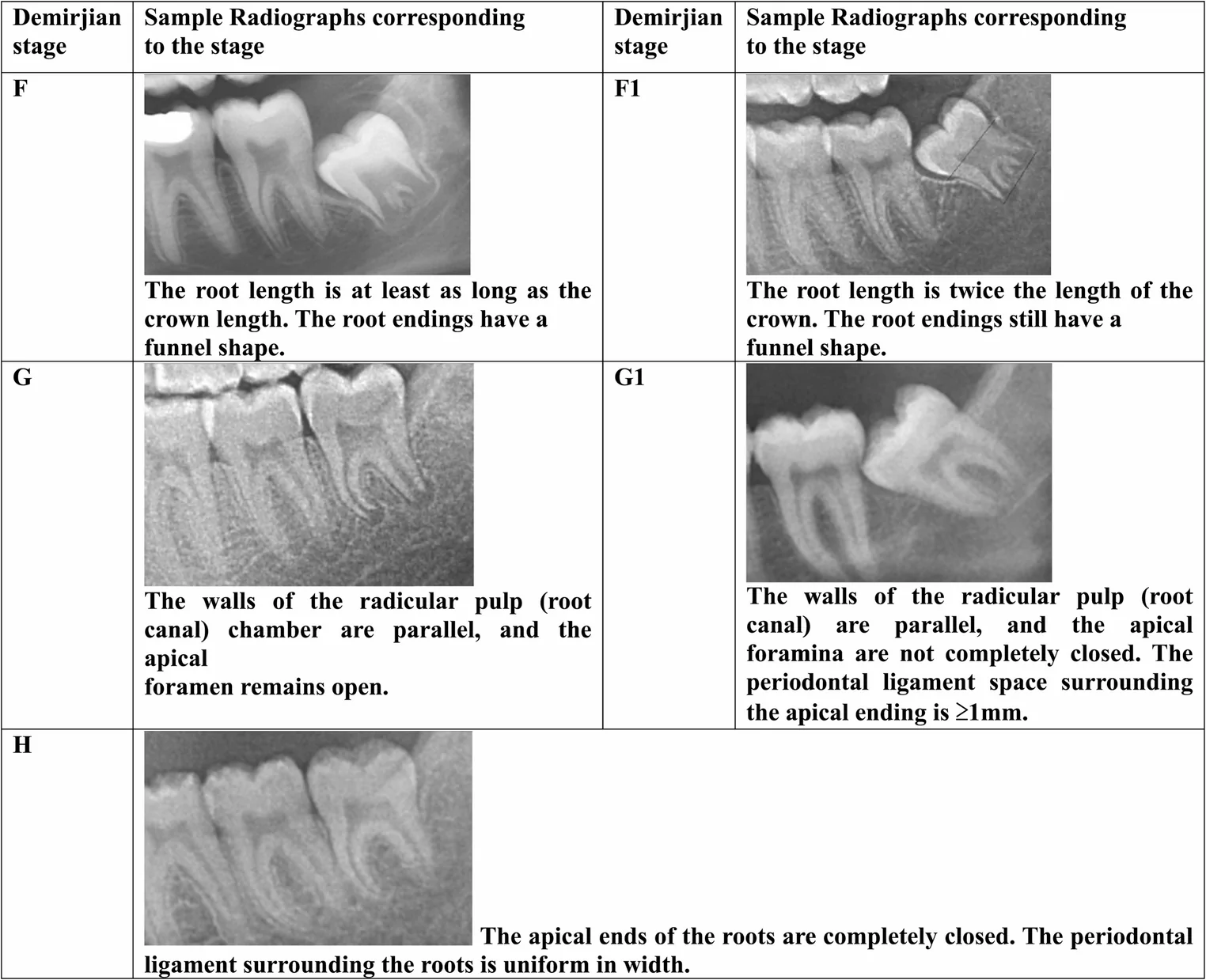
Demirjian third molar development staging F-H as modified by Solari et al. [16]

The maturation of the mandibular left third molar was initially assessed using the conventional Demirjian eight-stage classification (A–H) [6]. Following a one-month washout period to minimise recall bias, the same panoramic radiographs were re-evaluated using the Solari and Abramovitch modification of the Demirjian system [16], which incorporates two additional intermediate root stages (F1 and G1). Stage F1 is characterised by a root length approximately twice the crown length, with apices that remain funnel-shaped. Stage G1 is defined by parallel root walls with incompletely closed apices and a periodontal ligament space at the apex exceeding 1.0 mm (Fig. 1). In mandibular third molars exhibiting different developmental stages between mesial and distal roots, the less advanced root determined the final stage assignment [6, 22].

The first author performed all radiographic assessments. Intra-examiner reliability was evaluated by re-scoring a randomly selected 10% subsample of the radiographs (*n* = 60) after a two-week interval. Inter-examiner reliability was assessed by the second author, who independently evaluated the same subsample. Both examiners applied the conventional Demirjian staging system and the Solari and Abramovitch modification during reliability testing.

### Statistical analysis

All statistical analyses were performed using IBM SPSS Statistics (Version 29.0; IBM Corp., Armonk, NY, USA). Statistical significance was set at *p* < 0.05. Third-molar maturation served as the index test and was evaluated using three sequential cut-off criteria: stage G and above (≥ G), refined stage G1 and above (≥ G1), and stage H (complete apical closure). Each cut-off was treated as a binary diagnostic test, with stages at or beyond the cut-off classified as test-positive and earlier stages as test-negative. All analyses were conducted separately for males and females to account for potential sexual dimorphism in third molar maturation.

For each sex and each cut-off stage, 2 × 2 contingency tables were constructed to cross-classify third-molar stage (test positive vs. negative) against chronological age status (≥ 18 vs. < 18 years). These tables yielded counts of true positives (TP), false positives (FP), true negatives (TN), and false negatives (FN). The association between third-molar stage and age status was assessed using the Pearson chi-square test. Odds ratios (ORs) were calculated to quantify the strength of association between attainment of the specified third-molar stage and the likelihood of being ≥ 18 years. For each cut-off stage (≥ G, ≥G1, and H), and separately for males and females, the following diagnostic accuracy measures were calculated: sensitivity, specificity, positive predictive value (PPV), negative predictive value (NPV), accuracy, and likelihood ratios (Table 2).

**Table 2:** Summary of diagnostic measures and their interpretation in the context of third-molar maturation for identifying attainment of 18 years

Diagnostic measure	Description/ Calculation	What it represents	Interpretation
True positive (TP)	Individuals aged ≥ 18 years correctly classified as ≥ 18	Correct identification of legally adult individuals	Higher TP values indicate a better ability to detect individuals who have attained 18 years.
False Positive (FP)	Individuals < 18 years incorrectly classified as ≥ 18	Erroneous classification of minors as adults	Represents over-aging with significant ethical and legal implications.
True Negative (TN)	Individuals < 18 years correctly classified as < 18	Correct identification of minors	Minimises the risk of assigning adult status to under-18 individuals
False Negative (FN)	Individuals aged ≥ 18 years incorrectly classified as < 18	Failure to detect legal adulthood	Leads to conservative classification, generally considered safer in forensic contexts
Sensitivity (Sens)	TP/ (TP + FN)	Probability that individuals aged ≥ 18 years are correctly identified	High sensitivity reduces the likelihood of misclassifying adults as minors
Specificity (Spec)	TN/ (TN + FP)	Probability that individuals < 18 years are correctly excluded	High specificity limits false classification of minors as adults
Positive Predictive Value (PPV)	TP/ (TP + FP)	Probability that an individual classified as ≥ 18 (e.g. ≥G/≥G1/H) is truly ≥ 18 years	Provides case-level evidential support for concluding attainment of 18 years
Negative Predictive Value (NPV)	TN/ (TN + FN)	Probability that an individual classified as < 18 (e.g. < G/< G1/< H) is truly < 18 years	Indicates the reliability of the method in excluding legal adulthood
Accuracy (Acc)	(TP + TN) / Total	Overall correct classification rate	Summarises the combined discriminative performance
Odds Ratio (OR)	(TP × TN)/(FP × FN)	Strength of association between tooth stage and ≥ 18 status	Quantifies the evidential strength of the staging criterion
Likelihood Ratio Positive (LR+)	Sens / (1- Spec)	Degree to which the odds of being ≥ 18 increase following a positive test	Values > 10 indicate strong support for adult classification
Likelihood Ratio Negative (LR–)	(1- Sens)/Spec	Degree to which the odds of being ≥ 18 decrease following a negative test	Lower values indicate stronger evidence against the attainment of 18 years
Post-test Probability (Positive)	[Pre-odds × LR+] / [1 + (Pre-odds × LR+)]	Probability of being ≥ 18 years given a positive stage (e.g. ≥G/≥G1/H)	Reflects the evidential probability of adulthood after observing advanced third-molar maturation
Post-test Probability (Negative)	[Pre-odds × LR–] / [1 + (Pre-odds × LR–)]	Probability of being ≥ 18 years given a negative stage (e.g. < G/< G1/< H)	Indicates residual risk of adulthood despite an immature third-molar stage

To discriminate between individuals aged < 18 years and those who have attained 18 years, both pre-test and post-test probabilities of being ≥ 18 years were estimated. The pre-test probability (P_0_) represents the likelihood that an individual in a given population is 18 years or older before application of the diagnostic test, reflecting the underlying age distribution of the sample. In contrast, the post-test probability (PTP) is the probability that an individual is 18 years of age, given the result of the diagnostic test. Sex-specific pre-test probabilities were derived from the observed prevalence of individuals aged ≥ 18 years in the study sample (41.4% for males and 39.0% for females). Post-test probabilities were then calculated using Bayes’ theorem for each third-molar cut-off stage (G, G1, and H), separately for males and females. These estimates quantify the probability that an individual has reached 18 years of age given the observed third-molar maturation stage.

## Results

Intra- and inter-examiner agreement for Demirjian’s stages of third-molar development was assessed using Cohen’s kappa (κ) statistics. Overall, intra-examiner agreement was substantial (κ = 0.912), indicating excellent consistency in repeated assessments by the same examiner. Inter-examiner agreement was lower but remained within the substantial agreement range (κ = 0.731), indicating good reproducibility between observers when applying the staging criteria.

Stage-specific analysis revealed marked differences in reproducibility. Inter-examiner agreement was excellent for stages G (κ = 0.946) and H (κ = 0.967), reflecting high consistency in identifying advanced root development and complete apical closure. However, agreement on the modified stage G1 was poor (κ = 0.721), indicating difficulty in consistently distinguishing this transitional stage across examiners.

Tables 3, 4 and 5 show the cross-tabulation of third molar maturation stages and chronological age status. They demonstrated a strong association between third-molar maturation stage and chronological age status in both sexes for all evaluated cut-off stages (G, G1, and H). For males, the proportion of individuals classified as ≥ 18 years increased progressively with advancing third-molar development, with the highest number of true positives observed at stage G and above and the fewest false positives observed at stage H. A similar trend was observed in females, with a higher proportion of advanced stages below 18 years at earlier cut-offs, consistent with earlier maturation patterns.

**Table 3:** Diagnostic cross-tabulation of conventional Demirjian stage G against chronological age status (*≥* 18 years vs. < 18 years) in both sexes

	*≥* 18years	< 18 years	Total
Males			
Stage G & above (positive)	116^TP^	34^FP^	150
Stages A to F (negative)	04^FN^	126^TN^	130
Total	120	160	280
Females			
Stage G & above (positive)	146^TP^	31^FP^	177
Stages A to F (negative)	14^FN^	128^TN^	142
Total	160	159	319

***TP: True positives; FP: False positives; TN: True Negatives; FN: False Negatives***

**Table 4:** Diagnostic cross-tabulation of modified Demirjian stage G1 against chronological age status (*≥* 18 years vs. < 18 years) in both sexes

	*≥* 18years	< 18 years	Total
Males			
Stage G1 & above (positive)	113^TP^	33^FP^	146
Stages A to G (negative)	07^FN^	127^TN^	134
Total	120	160	280
Females			
Stage G1 & above (positive)	142^TP^	26^FP^	168
Stages A to G (negative)	18^FN^	133^TN^	151
Total	160	159	319

***TP: True positives; FP: False positives; TN: True Negatives; FN: False Negatives***

**Table 5:** Diagnostic cross-tabulation of conventional Demirjian stage H against chronological age status (*≥* 18 years vs. < 18 years) in both sexes

	*≥* 18years	< 18 years	Total
Males			
Stage H (positive)	102^TP^	24^FP^	126
Stages A to G (negative)	18^FN^	136^TN^	154
Total	120	160	280
Females			
Stage H (positive)	118^TP^	13^FP^	131
Stages A to G (negative)	42^FN^	146^TN^	188
Total	160	159	319

***TP: True positives; FP: False positives; TN: True Negatives; FN: False Negatives***

Sex-specific diagnostic accuracy measures for stages G, G1, and H are summarised in Table 6. In males, stages G and above demonstrated the highest sensitivity (96.7%), indicating excellent ability to identify individuals aged ≥ 18 years, while stage H showed a more balanced sensitivity and specificity (both 85.0%). Stages G1 and above provided intermediate performance, with a sensitivity of 94.2% and a specificity of 79.4%. Negative predictive values were highest for stages G and above and stages G1 and above, reflecting strong exclusion of adulthood when these stages had not been reached.

**Table 6:** Sex-specific diagnostic performance of third-molar maturation cutoffs (G, G1, and H) for identifying individuals aged *≥* 18 years

Cut-off (Positive test)	Sensitivity % (95% CI)	Specificity % (95% CI)	PPV (95% CI)	NPV (95% CI)	Accuracy (95% CI)	LR+ (95% CI)	LR- (95% CI)	Odds Ratio (95% CI)
Males (*n* = 280)								
Stage G & above	96.7 (92.1–98.6)	78.8 (72.0–84.3)	77.3 (70.0–83.2)	96.9 (92.7–98.7)	86.4 (81.8–90.0)	4.55 (3.37–6.14)	0.04 (0.02–0.11)	107.6 (35.1–329.8)
Stage G1 & above	94.2 (88.3–97.2)	79.4 (72.5–85.0)	77.4 (70.1–83.3)	94.8 (90.1–97.3)	86.0 (81.4–89.6)	4.57 (3.32–6.29)	0.07 (0.03–0.16)	62.2 (24.7–156.9)
Stage H	85.0 (77.5–90.3)	85.0 (78.4–90.0)	81.0 (72.8–87.2)	88.3 (82.3–92.4)	85.0 (80.5–88.7)	5.67 (3.87–8.32)	0.18 (0.11–0.28)	32.2 (15.8–65.6)
Females (*n* = 319)								
Stage G & above	91.3 (86.0–94.7)	80.5 (73.6–86.0)	82.5 (76.4–87.2)	90.1 (84.6–93.8)	85.9 (81.5–89.5)	4.68 (3.40–6.44)	0.11 (0.07–0.18)	43.0 (20.0–92.4)
Stage G1 & above	88.7 (82.7–92.7)	83.7 (77.0–88.7)	84.6 (78.4–89.2)	88.1 (82.2–92.2)	86.2 (82.1–89.6)	5.45 (3.82–7.78)	0.14 (0.08–0.22)	40.3 (21.0–77.5)
Stage H	73.8 (66.3–80.0)	91.8 (86.6–95.2)	90.1 (84.0–94.0)	77.7 (71.1–83.1)	82.1 (77.6–85.9)	9.02 (5.54–14.68)	0.29 (0.21–0.38)	31.4 (15.8–62.2)

*PPV*,* Positive Predictive value; NPV*,* Negative Predictive value; LR+*,* Positive Likelihood ratio; LR-*,* Negative Likelihood ratio*

In females, sensitivity decreased with advancing stage, from 91.3% at stages G and above to 73.8% at stage H. In contrast, specificity increased from 80.5% at stages G and above to 91.8% at stage H. Consequently, positive predictive value was highest at stage H in females (90.1%), indicating strong confirmatory value when apical closure was complete. Likelihood ratios supported these patterns: earlier cut-offs (G and above and G1 and above) yielded moderate LR+ values (approximately 4 to 5), whereas stage H provided strong diagnostic evidence in females (LR + = 9.02). Overall, accuracy remained high across all cut-offs in both sexes (approximately 82–86%).

Table 7 shows the sex-specific post-test probabilities, translating diagnostic performance into probabilistic events relevant for forensic interpretation. In males, a positive result at stages G and above, and at G1 and above, increased the post-test probability of being ≥ 18 years to approximately 76%, while a positive result at stage H increased this probability to 80%. In contrast, negative test results at stages G and above, and at G1 and above, reduced the probability of being ≥ 18 years to below 5% and stage H to 11%, indicating strong exclusion of adulthood when these stages had not been reached. In females, post-test probabilities following a positive test increased from 75% at stages G and above to 78% at stages G1 and above. They exceeded 85% at stage H, reflecting the higher specificity of advanced maturation in females. Negative test results reduced the probability of being ≥ 18 years to approximately 6–8% for stages G and above and G1 and above, and to 15.5% for stage H.

**Table 7:** Sex-specific post-test probabilities for *≥* 18 years (≥ conventional Stage G vs. conventional Stage H vs. ≥ modified Stage G1)

Sex	Cut-off (Positive test)	Pre-test P (*≥* 18)	Post-test P (*≥* 18/ Positive)	Post-test P (*≥* 18/ Negative)
Males	Stage G & above	41.4%	76.3%	2.9%
	Stage G1 & above	41.4%	76.4%	4.9%
	Stage H	41.4%	80.0%	11.1%
Females	Stage G & above	39.0%	74.9%	6.5%
	Stage G1 & above	39.0%	77.6%	7.9%
	Stage H	39.0%	85.2%	15.5%

## Discussion

A ten-stage scoring system was originally proposed to achieve greater resolution in characterising late root development as teeth approach apex closure [16]. Although Demirjian et al. did not define intermediate stages such as F1 and G1 within the permanent molar classification [6], subsequent authors have suggested that these additional stages may be particularly informative for assessing third-molar maturation [16]. The present study provides a direct forensic evaluation of this proposition by comparing the refined G1 stage with the conventional G and H stages for determining attainment of 18 years. Our findings demonstrate that, while the refined G1 stage offers modest gains in specificity and post-test probability, particularly among females, its overall diagnostic performance was broadly comparable to that of stage G and inferior to that of stage H in confirmatory contexts.

In the present study, we found that the conventional Demirjian stages showed the highest kappa scores for both intra- and inter-examiner readings for mandibular third molars. However, the inter-examiner agreement was moderate overall when the refined Demirjian stages were applied, with the greatest variability observed for the G1 stage. This finding is consistent with previous reports highlighting the interpretative challenges associated with intermediate root sub-stages [18]. Dhanjal et al. reported particular difficulty in distinguishing stages F1 and G1 from adjacent conventional stages, noting that observers often found the unmodified Demirjian stages (F, G, and H) easier to apply consistently, with higher reproducibility [4]. Similar findings were reported by De Salvia et al., who observed that identification of F1 and G1 introduced additional observer-related uncertainty and did not improve diagnostic accuracy, leading the authors to favour the original eight-stage system [23]. The moderate inter-examiner agreement observed for G1 in the present study likely reflects the subtle morphological criteria that define this transitional stage, namely, parallel root walls with incomplete apical closure and residual periodontal ligament space, which may be variably perceived depending on image quality and observer experience. Importantly, agreement for the conventional late stages G and H remained excellent, reinforcing their robustness and reproducibility in forensic casework. These findings underscore the importance of structured calibration and targeted training in stage assessment to minimise intra- and inter-observer variability, particularly when refined staging systems are applied [24]. Examiner experience, clarity of stage definitions, and the number of developmental categories employed are all critical determinants of reproducibility and should be carefully considered when implementing modified staging systems in medico-legal age estimation.

These observations are further supported by the South African study by Uys et al., which evaluated the modified Demirjian approach incorporating G1 and reported that the greatest inter-observer disagreement occurred at the G–G1 transition [25]. The present findings differ somewhat from those reported by Gupta et al., who observed very good inter-observer agreement for both the conventional Demirjian method (κ = 0.840) and the Solari and Abramovitch modification (κ = 0.843) [17]. However, their reliability estimates were reported for the staging systems overall rather than for individual developmental stages. In contrast, the present study identified the greatest observer variability at stage G1, suggesting that the subtle morphological features defining this transitional stage may be more difficult to interpret consistently. In our data, the refined G1 cut-off provided diagnostic discrimination comparable to stage G (Area under the curve, AUC ≈ 0.86–0.89 across sexes), yet reproducibility for G1 remained lower than for stages G and H. Taken together, these findings suggest that while stage G1 provides finer developmental resolution and supports probabilistic inference near the 18-year threshold, its potential benefit is offset by limitations in reproducibility between examiners.

In forensic age estimation, minimising misclassification errors at the legal age threshold is critically important, as false positives carry ethical consequences. In contrast, false negatives may affect legal responsibility [26, 27]. In the present study, false-positive classifications, in which individuals younger than 18 years were assigned to advanced stages, were predominantly observed at stages G and G1, reflecting the well-recognised overlap between late root development and chronological age in this period. False-negative classifications, in contrast, were more frequently associated with individuals aged ≥ 18 years who had not yet reached apical closure, particularly when earlier cut-offs were applied. Similar findings have been reported in an Indian study. Acharya reported that conventional Demirjian third-molar staging correctly classified individuals as < 18 or ≥ 18 years in approximately 73.2% of test subjects, with a higher rate of correct juvenile classification (85.7%) than adult classification (60%), indicating a substantial proportion of adults were misclassified as juveniles or vice versa [9]. Similar observations have been reported by Mincer et al., De Salvia et al., and Dhanjal et al., who noted overlap in third-molar development between individuals younger and older than 18 years. More recently, Gupta et al. likewise demonstrated that although late third-molar stages provide useful age-related information, biological variation in maturation limits perfect classification [4, 11, 17, 28]. Collectively, these findings indicate that some degree of misclassification around the 18-year threshold is unavoidable due to biological variability and highlight the need for cautious interpretation of third-molar staging in forensic practice.

The findings of this study should also be considered in the context of the recommendations issued by the International Interdisciplinary Study Group on Forensic Age Diagnostics (AGFAD), which advocate a multidisciplinary approach to age estimation and emphasise that no single developmental indicator should be used in isolation when assessing legally relevant age thresholds [29, 30]. The present results support the value of third-molar staging as a component of forensic age assessment while also highlighting the importance of probabilistic interpretation and acknowledgement of biological variability. The use of likelihood ratios and post-test probabilities in the present study aligns with contemporary recommendations to express age-estimation findings in terms of evidential strength rather than definitive age assignments [30].

The key strength of the present study is the direct comparison between conventional Demirjian stages (G and H) and the refined G1 stage, addressing an important evidence gap in the literature. Also, the diagnostic accuracy framework in this study, which goes beyond descriptive age–stage associations, enables the direct translation of the findings into probabilistic statements relevant to medico-legal decision-making at the 18-year threshold. Several limitations should be acknowledged. Firstly, the study was retrospective and based on OPGs obtained originally for clinical purposes, which may have introduced variability in image quality and positioning. Second, although overall inter-examiner agreement for refined staging system was substantial, stage G1 demonstrated greater observer variability than the conventional late root stages. This suggests that the subtle morphological features defining G1 may be more difficult to interpret consistently and reinforces the need for careful examiner calibration when applying refined staging systems in forensic practice. Third, the study relied on mandibular third-molar development alone, and the inclusion of maxillary third molars or multimodal age indicators may have improved overall diagnostic performance. Fourth, while post-test probabilities provide valuable probabilistic insight, they are inherently dependent on the prevalence of individuals aged *≥* 18 years within the study sample. Consequently, the post-test probabilities reported in this study may not be directly transferable to populations with different age structures, demographic characteristics, or ethnic backgrounds. Caution is therefore warranted when applying these estimates outside the population from which they were derived. Lastly, the oldest age groups were represented by fewer individuals than the younger age groups, particularly among males. While unlikely to have affected the primary age-status analyses, this may have influenced the precision of estimates for the most advanced developmental stages.

Future research should focus on validating the diagnostic performance of refined third-molar staging systems in larger and ethnically diverse populations. Particular attention should be directed towards transitional stages such as G1, which demonstrated greater observer variability in the present study. Advances in artificial intelligence and deep learning have shown promise in dental age estimation and may improve objectivity, consistency, and reproducibility of stage assessment by reducing observer-dependent variation and providing support for forensic age estimation [31, 32].

## Conclusion

This study evaluated the forensic usefulness of conventional and refined third-molar maturation stages for determining attainment of 18 years in a South Indian population. Third-molar maturation showed a strong ability to distinguish individuals at the 18-year threshold in both sexes, with diagnostic characteristics varying by developmental stage. The conventional Demirjian stages G and H showed high diagnostic performance and excellent reproducibility, supporting their continued use in medico-legal age assessment. The refined G1 stage provided finer developmental resolution and yielded diagnostic performance comparable to stage G, with modest gains in specificity and post-test probability in females; however, these potential benefits were constrained by reduced inter-examiner agreement. Collectively, the findings indicate that while refined staging may enhance probabilistic interpretation of late root development, its routine forensic application should be approached with caution unless supported by rigorous calibration and training. Conventional late-stage Demirjian cut-offs remain the most reliable indicators for assessing attainment of 18 years of age.

## Data Availability

No datasets were generated or analysed during the current study.
